# Creating mindful heroes: a case study with ninth grade students

**DOI:** 10.3389/fpsyg.2023.1091349

**Published:** 2023-05-09

**Authors:** Inês Nogueira, Mariana Reis Barbosa, Luísa Mota Ribeiro, Ioanna Xenophontos, Philip Zimbardo

**Affiliations:** ^1^Universidade Católica Portuguesa, Faculty of Education and Psychology, Research Centre for Human Development, Porto, Portugal; ^2^Department of Psychology, School of Humanities and Sciences, Stanford University, Stanford, CA, United States

**Keywords:** Heroic Imagination Project, heroism, mindfulness, empathy, bystander effect

## Abstract

The Heroic Imagination Project (HIP) aims to redefine heroism as a set of habits that anyone can achieve. Research findings on the psychological foundations of negative forms of social influence that can lead to bystander behavior are translated into tools that individuals can use in their daily lives. Habits of wise and effective helping behavior are learned, modeled, and encouraged through the training of the “heroic imagination.” According to the literature, practicing mindfulness can increase empathy, compassion, and prosocial behaviors. There is empirical evidence that compassion can act as a mediator between mindfulness and prosocial helping behaviors toward strangers, suggesting that mindfulness promotes this behavior and thus helps to overcome the bystander effect. With this hypothesis in mind, we created a program that combined mindfulness and HIP sessions. Five participants volunteered to participate in the “Creating Mindful Heroes” 9-week program. Throughout the sessions, they filled in a diary, and at the end of the program, they answered two feedback questionnaires. They were then invited to participate in individual interviews. The participants reported a positive overall perspective regarding the program, mentioning several improvements in their relationships with their family, peers, and others in society. Moreover, participants reported that the program promoted prosocial behaviors and aided them in developing empathy.

## Introduction

The Heroic Imagination Project (HIP) was founded by Philip Zimbardo to develop people's commitment to the construction and maintenance of transparent and open cultures that promote ethical behavior. HIP aims to understand the underlying psychological processes (e.g., the bystander effect) that lead to difficult social situations and unwanted behavior, as well as the ability to analyze and respond to social pressure effectively (Zimbardo, 2019). In Portugal, HIP trainers have adapted the program for teenagers and conducted a pilot study with 228 adolescents. Pre and post-test assessments using two self-report scales revealed that the intervention had a positive impact on the experimental group, as they showed higher prosocial attitudes and behavior when compared to the control group (Santo, 2018). According to Jones (2017), the research conducted on mindfulness has shown that its practice addresses several of the heroic traits identified by Franco et al. (2011), which can increase prosocial behaviors (Luberto et al., 2018). With this hypothesis in mind, we decided to create a new program (Creating Mindful Heroes) based on the HIP curriculum with an added mindfulness component. We also aimed at exploring participants' perspectives and experiences with and during the program, as well as how they perceived changes in their relationships with family members, peers, and others in society.

### From the banality of evil to the banality of heroism

In the 70s, Philip Zimbardo conducted a study at the University of Stanford that replicated a prison environment with the main objective of researching how readily participants who were randomly assigned to guard or prisoner roles would conform to these roles (Zimbardo, 2007). The results of this experiment demonstrated the influence of situational variables on individual behavior, revealing that situational forces can influence and pervert individual dispositions (Haney et al., 1973; Zimbardo, 2007). Arendt's concept of Banality of Evil, which suggests that ordinary people can commit unimaginable evil acts under certain conditions and social pressures, agrees with the Prison Stanford Experiment (Zimbardo, 2007, p. 288; Blau et al., 2009). Franco and Zimbardo (2006) created the opposite concept, the Banality of heroism. Heroism suggests that everyone has the potential to be a hero and that it is possible to change people's mentalities so that they help others, have compassion for others, and develop confidence in their ability to act heroically. According to Franco et al. (2011, p. 101), “*Heroism is a social activity: it implies a service to others, be it a person, group or community, or in the defense of an ideal, socially sanctioned or by searching for new social norms, it implies a voluntary commitment and the recognition of possible risks / costs that the individual is willing to accept, without expecting any reward*”. Despite an almost overwhelming interest in the psychology of evil (Franco and Zimbardo, 2016), the phenomenon of heroism has only recently gained serious relevance in multiple fields, especially psychology, becoming increasingly data-driven in the last decade (Franco, 2016; Franco et al., 2016). However, little is known about whether we can be trained as children or adults to think and behave heroically in predictable ways (Slagter et al., 2011; Jones, 2017 cited in Jones, 2019). Kohen et al. (2017) suggest four trainable skills: developing a heroic imagination about how you would act; cultivating a sense of empathy and commonality with others; practicing small-scale helping habits; and acquiring heroic-specific skills or traits. The Heroic Imagination Project is based on the idea that an individual who has already considered scenarios requiring heroic action (and the personal consequences thereof) is more likely to act heroically when the opportunity arises (Blau et al., 2009).

### Counteracting bystander behavior through the heroic imagination

There are, however, various obstacles to heroism, so in order to develop the heroic imagination, the individual must also learn to anticipate potential obstacles to heroic action. Therefore, training the heroic imagination entails trainees being aware of the psychological foundations of negative forms of social influence that can lead to bystander behavior. Since the murder of Kitty Genovese in 1964, social psychology has been investigating what causes people to intervene in dangerous situations (Piliavin et al., 1969). According to Fischer et al. (2011), Kitty Genovese's inaction was mainly due to three distinct psychological processes. Diffusion of responsibility explains how, in a situation with many bystanders, responsibility is divided among them, resulting in a delay in helping the person in need or not acting at all. Concerning evaluation apprehension, this argues that people are afraid of intervening because they will make mistakes or act incorrectly, resulting in inaction. Finally, pluralist ignorance occurs when people rely on others' reactions to determine whether or not they are in an emergency situation. Literature have also shown the role of race in bystander behavior (Gaertner and Dovidio, 1977; Katz et al., 2018 cited in Williams et al., 2022). When someone witnesses a morally wrong behavior, they are more likely to intervene based on the extent of the unfairness, and they will react based on their personality and their feelings about the community (Moisuc et al., 2018). However, when people learn to distinguish themselves based on their race, it can be challenging to cognitively understand and act on the unfairness. For example, Williams et al. (2022) contend that White people are socialized to ignore social injustice. The role of socialization in bystanderism is crucial because studies show that children at early ages act on the impulse to help without being concerned about the rules (Hepach et al., 2012; Beier et al., 2014 cited in Williams et al., 2022). Staub's (1971) research findings revealed that this natural inclination to help increases until the second grade remains stable until the fourth grade, and then declines in the sixth grade until the level of kindergartens. This seems to be related to the learning of conventional rules of behavior, as children of this age would stop helping (in Staub experiments) because they were afraid of breaking the rules. When a potential helper perceives some incompatibility between “appropriate” behavior and helpful behavior, adherence to these rules may inhibit attempts to help others (Staub, 1971). It seemed that children learned conventional rules of behavior and were not taught that under certain circumstance the need of another person for help overrides such rules (Staub, 2019). These results highlight the need to teach children to discriminate between situations in which the usual rules of appropriate social behavior apply and those in which the need to help others supersedes them, in order to decrease the bystander behavior due to disapproval fear (Staub, 1971).

### Mindful-based heroism

Jones (2017, 2019) demonstrates that there are considerable similarities between mindfulness and heroism. Mindfulness emphasizes the wellbeing of all, advocating the dissolution of ego grasping, which should be accompanied by daily altruistic sacrifice toward people and the world. In heroism, there is a type of quest, a risk, or sacrifice (whether they are short or long term), and one can choose to take an active or passive role. In the same study, the author identifies several results from practicing mindfulness that improve important characteristics of a hero, such as focused attention, awareness of the present, spontaneous kindness, better attentional functioning, increased cognitive control, more efficient conflict monitoring, decreased alert response, the perception of ego grasping as illusory, having no sense of self, improved primary sensory awareness, and finally an increase in empathy (Jones, 2017).

Luberto et al. (2018) meta-analysis supports the efficacy of MBIs when it comes to the increase of empathy, compassion, and prosocial behaviors. These interventions showed great improvements in at least one prosocial behavior when compared with control groups. They also found out that meditation training had a small-medium significant effect on prosocial outcomes (with a higher number when there was an active control group). This increase in empathy is corroborated by many studies (Beitel et al., 2005; Dekeyser et al., 2008; Greason and Cashwell, 2009; Berry et al., 2018), proposing that individuals, by practicing decentering (Shapiro et al., 2006), will be more aware of the present and therefore more aware of others' mental states. Given that the individuals are more in tune with their emotional states, they are more understanding of the emotions of others (Block-Lerner et al., 2007). Regarding emotional mechanisms that are related to meditation that can lead to more prosocial outcomes, they include: increased positive affect, a sense of a socioemotional connection with others, improved mindfulness and self-compassion traits, and a noticeable decrease in stress and negative affect (Shapiro et al., 1998; Hutcherson et al., 2008; Oman et al., 2010; Kok et al., 2013; Wallmark et al., 2013; Kang et al., 2014; Ashar et al., 2016).

In the study of Lim and DeSteno (2016), the authors found that more empathy can lead to more compassion, which leads to greater prosocial behaviors. The study by Berry et al. (2018) also showed that compassion (empathic concern) was a mediator between mindfulness and prosocial helping behaviors toward strangers, suggesting that mindfulness promotes these behaviors and helps in overcoming the bystander effect. These results are also corroborated by the existing literature (Shapiro et al., 1998; Hutcherson et al., 2008; Kok et al., 2013; Kang et al., 2014; Ashar et al., 2016).

## Method

### Objectives

This study intended to create and implement a new program for high school students that incorporated mindfulness techniques into the HIP curriculum, called Creating Mindful Heroes (CMH). We also aimed to explore their perspectives of various contexts, such as their family and school lives, as well as on and during the CMH sessions.

These were the following specific objectives defined:

Analyze participants' feedback on the program;

Analyze participants' knowledge after going through CMH;

Analyze participants' intrinsic experience throughout the program's sessions;

Analyze participants' intrinsic experience throughout the program in general;

Analyze participants' perception regarding changes in their relationships toward others in society;

Analyze participants' perception regarding changes in their relationships toward their family members;

Analyze participants' perception regarding changes in their school life.

### The program

The Creating Mindful Heroes program included nine sessions in total, five of which were dedicated to mindfulness and three of which were adapted from the original Portuguese Heroic Imagination Project program (Table 1). The sessions alternated between mindfulness and HIP contents and lasted for 9 weeks, lasting 1 hour each. The mindfulness sessions of the curriculum were delivered by a trained professional and instructor for the program Still Quiet Place (Saltzman, 2016). All the contents were written in English.

**Table 1:** Description of the Creating Mindful Heroes program sessions.

**Session**	**Objectives**	**Activities**
1 Mindfulness	• Understand the definition of Mindfulness • Learn about some the benefits of practicing mindfulness • Learn how to do breathing-based meditation	• Define Mindfulness • Discuss the benefits of practicing Mindfulness • Finished the session by doing breathing-based meditation • The participants were invited to download the “Headspace” app for their cell phone
2 Mindfulness	• Revision of what was learned in the last session • Initial discussion about the attitudes of a mindful person • The beginner's mind • Letting go	• Discussion about what was learned in the last session • Discussion of what is a beginner's mind • Discussion of what is letting go • Breathing-based meditation • The participants were encouraged to practice these two mindful attitudes that were presented in the session
3 HIP	• Presentation of what is the Heroic Imagination Project (HIP) • Discussion and explanation of what is a hero • Discussion and explanation of what is the Bystander Effect	• Introduction to HIP • “What is a hero” dynamic (With the help of post-it notes the student was asked to describe what a hero was to them and then present it to the rest of the class while placing it around a human figure) • Discussion of the before-mentioned dynamic (The students were invited to explain their choices) • Introduction to the Stanford Prison Experiment and how it developed into HIP • We started the discussion about the concept of the bystander effect • The students were invited to reflect and to share in the next HIP session a situation where they were a bystander
4 HIP	• Continuation of the discussion about the Bystander Effect • Discuss the effects of conforming • Discuss the five obstacles that prevents someone from helping	• Remember what was discussed in the last session • Discussion with the students about their previous experiences being bystanders (They were then invited to explain why they didn't help) • Deeper discussion about the bystander effect with the help from a video clip that showed various situations in which people were being part of social experiments in the streets of London (While watching the video strategic pauses were made so that the students could comment about how they were feeling, what they thought what the Bystanders were thinking and why they didn't help the people in need) • Discussion about the topic about conforming the five obstacles • Example of the Asch's conformity experiments where three of the students were stooges and the two remaining students were experimental participants (The image used for the example was taken from the internet and the three students knew before the session what their role was) • A video was watched as a way of summarizing the dangerous effects of conforming – this was an experiment where smoke came from the door of room • A more in-depth discussion was had about the five obstacles to helping: Diffusion of responsibility; The spotlight Effect; Group Ignorance; You're distracted/in a hurry; Helping could be dangerous • It was suggested to the students to engaged in a daily challenge during the next 15 days of Easter Holidays which the end product would be a photo album (physical or digital) with a brief description of how they completed the challenge
5 Mindfulness	• Discussion about what Active Listening is • Understand what it is and how you do it • Discussion about the non-verbal behaviors from active listening	• The session started by doing a dynamic activity where the students were separated into pairs, where one of was the listener and the other the speaker and after three minutes the roles would switch. The goal was that the listener didn't talk (e.g. couldn't ask questions) and couldn't show any type of judgement. The speaker had to talk about a personal subject • It was then discussed about the student's personal experience in each role • Discussion about non-verbal communication with emphasis about the non-verbal communication during active listening • Breathing-based meditation
6 Mindfulness	• Continuation of the objectives from last session • Discussion about non-violent communication	• Continuation of the active-listening discussion • It was then introduced the topic of Non-violent Communication • Body-scan meditation
7 HIP	• Discuss the topic of discrimination (A special focus in the events during the Holocaust and during Apartheid) • Discuss how these events relate to the topics already discussed before such as the bystander effect and conformity	• Video about the Blue Eyes Experiment • Discussion about why this experiment exists, how it was done and the ethical issues behind it • Relate this experiment to the phenomena that occurred during the Stanford Prison Experiment and the concepts learned about the bystander effect • Watching the movie “The Wave (1981)” • Discussion of the film, relating it with the concepts learned previously
8 Mindfulness	• Discuss the concept of gratitude • Relate this concept with empathy and mindfulness • Introduction to the Loving-kind meditation	• The students were asked to write a list about what they were grateful for • Discussion of what they wrote and how gratitude is the base to our being • Relate gratitude with empathy • Discussion about empathy and its relationship with mindfulness • Exercise of the loving-kind meditation. Example: “I am happy because the others and all living things are happy”
9 Mindfulness and HIP	• Conclusion of the CMH programme • Summary of all the concept learned throughout the programme • Discussion about what an Enlightened Hero is. Relate the concepts taught in the HIP sessions with those taught in the Mindfulness sessions	• Summary of the concepts learned throughout the Mindfulness and HIP sessions • Discussion about the relationship between these • Conclusion of what is an Enlightened Hero • The students were encouraged to promote prosocial and altruistic behaviors and that they were now setting the “example” of a hero

The teacher's role was that of a mediator, coach, and guide, in which we would give prompts to the participants and attempt to engage students in discussions.

### Participants

The recruitment of participants was done by a 15-min presentation in a private high school. Five female students from different nationalities volunteered to participate. The participants were between the ages of 14 and 15 (*M* = 14.20, *SD* = 0.45). Participants were asked if they had prior knowledge about either mindfulness or HIP. Four participants reported knowing about mindfulness as opposed to one who said they were unaware. Four participants reported that they did not know about HIP, while one participant reported that she did know about it. We also asked participants if they had previously practiced mindfulness, and one of them had.

### Materials

#### Socio-demographic questionnaire

In the socio-demographic questionnaire, participants were asked about their age, nationality, gender, which form class they were in and their parents' education level. They were then asked to answer about their awareness of HIP and mindfulness before the program presentation. Finally, they were asked if they had ever practiced mindfulness.

#### The creating enlightened heroes diary

This diary was given in a booklet format to the participants at the end of each session. Here, the participants were encouraged to write freely about their experiences during the session (feelings, thoughts, lessons learned, and personal changes they had noticed). To preserve their anonymity, they were asked to identify their diaries with the same code as their socio-demographic questionnaire.

#### Feedback questions

At the end of the program, we asked participants to answer two questions regarding their opinion about the CMH. The first question was: “What did you like most about the Creating Mindful Heroes programme?” and the second question was: “Where do you think we can improve and how can we achieve this?”.

#### Individual interviews with the students

The dimensions explored with the participants were their program feedback and their knowledge of the content taught during both the mindfulness sessions and the HIP component. The script also included questions regarding the participants' intrinsic experiences, their relationships with society, their school lives, and their relationship with their families. Individual interviews were scheduled 2 weeks after the CMH and were expected to last ~15 to 20 min.

### Procedure

The implementation of the CMH program started after the school granted us permission to start. We carried out this study following the recommendations from the ethics committee at the Catholic University of Portugal. All participants and their guardians provided written informed consent to participate in the research in accordance with the Declaration of Helsinki. Participants were informed about the objectives of the study, and their confidentiality and anonymity were assured (e.g., having pseudonyms). Participation was voluntary, and researchers informed students about data usage.

The participants filled in a socio-demographic questionnaire a week before the beginning of the study. Following this, participants started CMH with its 9-week curriculum. After each session, participants wrote in their Creating Mindful Heroes Diary. After the CMH was over, a feedback questionnaire was given to the participants. To match the responses from each participant in the different materials while maintaining the confidentiality and anonymity of the participants, the following pseudonyms were given: Maria, Carolina, Julie, Lucy, and Marta. Two weeks after the end of CMH, we requested participants to take part in individual interviews.

The data analysis, with NVIVO, followed a semi-inductive logic. We would then describe and, in some cases, quantify the phenomena that stood out from the participants' speeches (Elo and Kyngäs, 2008). Our unit of measurement was the idea of the participant, and through the process of open codification, major categories started to emerge. These were: descriptions, evaluations, evolution, experiences, learning outcomes, and level and session.

## Results

### Feedback from the program

Regarding participants' feedback on the CMH program, positive experiences included satisfaction, fun, interest, relaxation, expectation, and surprise. Some of the positive aspects of CMH mentioned include perceived changes in themselves as well as new ways to cope with daily issues “*I do think it does help people, who like me, are stressed out and can't think properly during stressful times..*.” (Carolina); the diversity of the curriculum was praised as well “*I liked how diverse the topics were and how they all interconnected and how the mindfulness played into the topics as well..*.” (Lucy). There were also mentions of overall satisfaction with the CMH program as a whole “*I hope that I will be able to join Mindful Heroes next year, because I had tons of fun this year!*” (Maria).

Although negative feedback was less prominent, all participants mentioned it at least once. They reported experiences of discomfort, sadness, difficulty, and preference for something else “*Sometimes when I meditated I would go too deep into my thoughts and then I would feel... sounds like a bit of a baby-ish thing to do but I would feel like crying because... like I don't know... I just felt like I delved into a place in my mind where I shouldn't have been, and I feel like crying… but I never know why*” (Maria).

### Knowledge acquired after the program

Marta, Maria, and Julie spoke about the *Beginner's Mind*, which is the ability to see everything as if it were the first time, freeing ourselves from expectations (Kabat-Zinn, 2003) “*Whenever you are doing anything you should be thinking about it… you should try to dedicate yourself like that scene in Ratatouille where he eats the cheese and he sees the things, he's not thinking about taxes or whatever… he's thinking about the cheese and describing it.”* (Maria). In contrast, Lucy and Marta describe *Letting Go* which is defined by Kabat-Zinn (2003) as accepting things just as they are, letting go of your expectations of them “*maybe it helped me not care as much, because I tend to become very sad when we fight, maybe it helped me not get as sad... move on, not overthink…*” (Marta). Regarding the active listening activity from session five, all participants were able to discuss and explain their take-away message (n = 5). Maria in particular stated that “*It was that we can identify a lot of how a person thinks just by looking at their face just by looking at their eyebrows the way they even their nose contorts…*”. Lucy concluded that the exercise was “*helpful on understanding how I can be more attentive during conversations*”.

When asked what the HIP's concept of a “everyday hero” was, all participants were able to answer. They mentioned that being a hero is not the opposite of being a villain, but of being a bystander, helping others in undesirable circumstances or even preventing these situations from occurring “*Somebody that does something every day that causes something good in the world, for example being a firefighter, doctor/ nurse or just being actually just a good person trying to help random people..*.” (Carolina).

Most of the participants were able to voice their thoughts on the relationship between the two components of the CMH. According to Julie, mindfulness helps fight the bystander effect by having a non-judgmental attitude toward the person in need, maintaining the helper's integrity “*I mean many times we do stuff we become bystanders because we don't like a person or we just feel uncomfortable in a situation and then mindfulness it helps us to be more neutral about all those things, just to accept things as they go and we know that what's right is right and what's wrong is wrong no matter the context or the situation*”. Lucy mentions that the mindfulness component helps keep attention to your surroundings, increasing the probability of helping behaviors occurring “*It's possible to be a hero without being mindful but you have to still pay a lot of attention, (…) but by being mindful in the moment you actually notice a lot more what people are doing around you and the effects that certain things will have on those people, and so by being mindful you can notice a lot more situations where you can be a hero, or at least help others be a hero*”. In a similar fashion, Carolina mentions that the relationship is linked to attention and our surroundings “*getting people to truly see how the world is and start focusing on their surroundings with what is happening around them…*”. Marta says that both components are related because they both help with self-improvement “*it all has to do with becoming a better person and giving more value to life!*”.

### Intrinsic experience regarding the program

With this objective, we aimed at exploring participants' experiences of the program as a whole, reporting personal changes that they noticed. The more predominant experiences reported by the participants were awareness (n = 5), calm (n = 4), and empathy (n = 5).

Regarding awareness, participants reported situations where they developed a mindful awareness “*It made me more perceptive of what's around me…*” (Carolina), “*when I used to be walking it used to just be like ‘Oh, I'm going to get to a certain place and be done with it' but with mindfulness I pay more attention to my surroundings and what's happening like as I'm going to the place*” (Julie), as well as bringing awareness regarding their personal responsibility “*It made me feel more responsible.*.” (Marta). Lucy reports that she started noticing more diffusion of responsibility in the corridors at school, and she says that by being aware she can now start trying to find ways to stop it from occurring “*but because of the CEH project I can at least see that it is happening, so I can try to learn how to find ways to go against that*.” Similarly, Maria says that the CEH program brought awareness to daily events and her impact on them “*I think that it alerted me a lot to what I see every day that I could change or have a positive impact (...)*”.

When evaluating the participants' empathy, we chose to take a multidimensional approach as suggested by Davis (1983) and created two categories utilizing their definitions for “Perspective Taking” (PT) and “Empathic Concern” (EC). The former refers to a more cognitive component, where the participant tries to understand the other's point of view and behavior, whereas the latter deals with the emotional aspect of the concept and refers to prosocial feelings of compassion and concern for the other. Regarding the PT component, Carolina, Lucy, and Maria mentioned this throughout their reflections regarding the bystander effect “*I feel like a lot of us can relate to that, because we don't do anything because we don't know why is it there (...)*” (Carolina), regarding how they now deal with people they do not like “*trying to be thankful for what they have done and what they could possibly do, that was actually very interesting and made it a lot easier to see they do have their own story (…) we don't really know what's going on in your life*” (Lucy), or even when explaining why they started greeting people on the street “*I always thought that at their time [the elderly people] specially in this country people were always like* “*Bom dia, bom dia [Good morning, Good morning!]” and nobody does that anymore I thought that they didn't really seem that happy for that and so I say good morning (...) and it's fun!*” (Maria). Regarding EC, Carolina, Julie, Maria, and Marta mentioned this while reflecting on the contents shown in the HIP sessions “…*that video that you showed us of people passing by a stroller but there was no baby in it but imagine if there was and people just pass through it and nobody's doing anything about it?*” (Carolina) and when they reflected why they helped someone else “*because I used to get bullied so there were a lot of people who were bystanders and let's say I on the other hand try not to be a bystander as much as I possibly can”* (Julie).

### Changes in interpersonal relationships

Participants reported that the CEH program helped them in their relationships. Julie reports that she noticed a positive difference in her relationship with her father “*I would say my relationship improved with my dad (...) we used to have a really close bond, but that broke off years ago, but sometimes I text him and everything so it's better…”* she further explains that the program helped reciprocate the effort to construct her relationship with him “*He himself has changed the way he treats me and I guess with mindfulness and everything I felt like, okay maybe I should give some of that back*”. A development in her empathy toward her father is identifiable, especially regarding perspective taking.

Lucy reported feeling more empathic, taking her sister's perspective while trying to understand her actions “*my sister and I don't always get along… I mean nine [years old] or so there's a big difference between us… but I am trying to see that she is nine, I can't expect her to act like a 12-year-old, (…) so taking in her personality and her story*”.

Lucy reported that as a result of CMH, she gained the confidence to successfully ask her friends to stop gossiping about others: “*sometimes my friends when they are right there around me they have stopped talking about certain things(...) I don't know if they still talk about it or not, but I have noticed that people do treat me differently..*.”. Maria reports that she has started empathizing more with her friends and that she now thinks about the different reasons for their actions “*since the program started I have been able to notice what they're thinking what they're feeling better, I can notice better the messages and hints they try to give me and I can think about their actions in many different ways*”.

Julie perceives that her attitudes toward her friends have changed as well, mentioning that she has become more caring, patient, and empathic with them “*the sessions had a lot to do with it because the constant saying of “you have to be more patient you have to think about it on the other person's perspective whenever you're mad, just breath” because we say stuff we don't want to and all that and I know deep down that my words can hurt them a lot*”.

Julie reports changes in her relationships with her colleagues as well as with strangers, stating that the program helped her integrate better with her form class by bringing her awareness to her isolation, and thus she was able to establish more acquaintances, especially with her colleagues, feeling reciprocated “*I speak to my classmates more (...) I try to help them more and they seem to confide more in me…”*. Regarding strangers, she describes a hypothetical experience of perspective taking when compared to her old habits “*if I see people are rude I just kind of ignore it and try to be as nice as possible or as unresponsive as possible… I try not to complain because they might be having a hard day or whatever, so I just go along my way*”.

Lucy reports feeling less socially awkward due to important social skills learned during the program that has benefitted her in other relationships “*I am getting there I am starting to help people more in the corridors if I see them fall I'll help them up..*.”.

Maria reports a change in attitude toward other people since she started the program “*the programme has helped me know that there are other things to worry about and every time I worry about something trivial, I always think, “wait a second! somebody was stabbed and nobody reported that!” so I think that it could have been in a much worse place than me*!” Regarding her interactions with strangers, she describes an experience of perspective taking when she explains why she now greets more the elderly on the street and that she has started complementing strangers more in the street “*I've also been complimenting strangers more it might seem like a weird thing to do, for example there was this lady, she had really cool lobster earrings and I was like “those earrings are really cool” and she said, “aw thanks!”*”.

Marta reports that the CMH program helped her overcome stereotypes that she had about her colleagues “*it helped me understand that the ones who we think are nerds and that I didn't get along with, can also be fun!*”.

## Discussion

The results demonstrate that the participants understood the benefits reported by Jones (2017, 2019) regarding their mindfulness sessions and its facilitating relationship with heroism, such as increasing their awareness and aiding their non-judgmental attitudes toward others. According to Shapiro et al. (2006), the practice of mindfulness facilitates a shift in perception where the participant can step away from their present experience. This, in turn, will make participants more aware of other's conditions, ultimately increasing one's empathy. Furthermore, MLERN (2012) report states that contemplative practices will promote empathy, empathic concern, problem-solving, and compassion, as evidenced by participants' reported empathy and situations in which people needed help. The results by Sanger et al. (2018) also further expose that participants who went through a mindfulness program reported being more aware of socially relevant emotional stimuli (regardless of their valence). These changes regarding others, colleagues, and friends were also seen in a group of participants who reported improvements in implicit attitudes toward more stigmatized groups by practicing loving-kindness meditation (which our participants also got to practice; Kang et al., 2014). Here, we can also observe the relationship between mindfulness and heroism, where all the components (better attentional functioning, more efficient conflict monitoring, spontaneous kindness, and increases in empathy) coalesce and improve relationships and, through it, stimulate with that, more prosocial behaviors (Jones, 2017; Luberto et al., 2018). This can be further explained by Eisenberg et al. (2006), that prosocial behaviors are linked to positive personal and socioemotional variables such as perspective-taking empathic concern, and positive peer and parental relationships, which these traits can be observed by what the participants report in this section as well in the intrinsic experience throughout the program.

In this study, we utilized a variety of materials to record data, and because it was qualitative, we were able to explore participants' experiences, perceptions, and perceived changes in a more profound way. The heterogeneous nature of our sample, particularly concerning nationalities, gives us a myriad of different perspectives and cultures, which ultimately enrich our data. This last strength can also be a limitation, as there can be a potential language barrier—though all participants spoke English, there was considerable variation in their level of fluency. Additionally, the small sample size, in conjunction with participants' gender, could have resulted in biases in their experiences and reflections. For instance, the literature reveals that compared with boys, girls tend to participate more in defending behavior (O'Connell et al., 1999; Pozzoli and Gini, 2010; Espelage et al., 2012; van der Ploeg et al., 2017 cited in Deng et al., 2021). Given that our sample only included girls, this moderating effect of gender was not analyzed. Having the participants' feedback in mind, other possible weaknesses concern the overall short length of the sessions, the imbalance in the distribution of the number of HIP sessions and mindfulness classes and, finally, the existence of only a single session in which both contents merged. Given Jones' (2017) hypothesis that the relationship between mindfulness and heroic traits can help build a more predictable and lasting hero, a limitation of this study is the lack of follow-up data. In order to fully grasp the benefits of incorporating mindfulness in HIP, this exploratory study should be followed by a randomized control trial that would allow the comparison between the outcomes of the HIP program and the outcomes of the CMH program, including pre, post, and follow-up measures.

## Conclusion

In conclusion, all participants reported a positive perception regarding the CMH program, with various improvements in participants' relationships (family, peers, colleagues, and others in society), and an increased awareness of the occurrence of prosocial attitudes and behaviors.

This was a pilot study where we attempted to successfully incorporate mindfulness with the HIP curriculum, as a response to the Jones' (2017) theoretical paper, as a way of showing that heroical qualities can be trained to promote prosocial behaviors. We hope that this can be the first stepping stone to more research regarding the field where heroism and mindfulness merge.

## Data availability statement

The raw data supporting the conclusions of this article will be made available by the authors, without undue reservation.

## Ethics statement

The studies involving human participants were reviewed and approved by Ethics Committee of Universidade Católica Portuguesa. Written informed consent to participate in this study was provided by the participants' legal guardian/next of kin.

## Author contributions

IN, MB, LR, and PZ contributed to the conception and design of the work. IN, MB, and LR were responsible for data collection, analysis, and interpretation of data. IN wrote the manuscript with valuable inputs from the remaining authors. IX and PZ made important intellectual contributions to the paper. All authors agreed on all aspects of the work and approved the version to be published.
